# Community structure, seasonal variations and interactions between native and invasive cattle tick species in Benin and Burkina Faso

**DOI:** 10.1186/s13071-016-1305-z

**Published:** 2016-01-27

**Authors:** Abel Biguezoton, Safiou Adehan, Hassane Adakal, Sébastien Zoungrana, Souaïbou Farougou, Christine Chevillon

**Affiliations:** Unité de Recherche en Biotechnologie de la Production et de la Santé Animales (URBPSA), Laboratoire de Recherche en Biologie Appliquée, Ecole Polytechnique d’Abomey-Calavi, 01 BP 2009 Cotonou, Bénin; Unité de Recherche sur les bases biologiques de la Lutte Intégrée (URBIO), Centre International de Recherche-Développement sur l’Elevage en zone Subhumide (CIRDES), 559, 3-51 Avenue du Gouverneur Louveau, 01 B.P. 454 Bobo-Dioulasso 01, Burkina Faso; IRD, UR 224 ‘Maladies Infectieuses et Vecteurs: Ecologie, Génétique, Evolution et Contrôle (MIVEGEC), Montpellier, France; Département des Sciences et Techniques de l’Elevage (DSTE/FASE), Université Dan Dicko Dan Koulodo, BP 465 Maradi, Niger; CNRS, Université Montpellier, UMR 5290 MIVEGEC, Montpellier, France

**Keywords:** Ixodidae, Biological invasion, Cattle health, *Amblyomma variegatum*, *Rhipicephalus microplus*, Community structure

## Abstract

**Background:**

The variation of tick abundance on ruminants had received little attention in West Africa before *Rhipicephalus (Boophilus) microplus* started to invade this region in the early 2000s. Ten years later, *R. microplus* was suspected to have replaced the native ticks. In addition to testing this hypothesis, this study investigated the interactions between native and invasive ticks and the relative role of climatic and geographical variables in the variations of tick community composition (beta diversity) on cattle herds.

**Methods:**

A one-year-long survey was performed in Benin and Burkina Faso during which adult ticks were collected from 144 steers from 12 localities in four different areas once a month. Morphological features were used to assign the collected ticks to different species (*A. variegatum*, *R. annulatus, R. decoloratus, R. microplus* and *R. geigyi)*. Beta diversity analyses and generalized linear models allowed characterizing the geographical variations in species assemblage and the effect of co-infestation patterns on the seasonal variations in the abundance and incidence rates of each taxon.

**Results:**

About 68 % (22,491/32,148) of all the adult ticks collected in one year were *R. microplus*. The most heterogeneously distributed taxa were *Hyalomma spp* and *R. microplus* and the lowest specific diversity was found in Central Burkina Faso. Although climatic variables did not provide any additional information on the variation in species assemblages compared with the sampling geography, adult tick abundance tended to peak during the late (*Boophilus* subgenus) or early (other taxa) rainy season. In most taxon-per-locality analyses, the abundance and incidence rate of a given tick taxon significantly increased when the host was co-infested by other taxa. The comparison with previous estimates (when possible) did not support the hypothesis that *R. microplus* invasion led to a decrease in native tick species abundance.

**Conclusions:**

The co-infestation patterns among native and invasive tick species are key factors for the determination of the community structure and the infestation dynamics of each tick taxon in West African cattle.

**Electronic supplementary material:**

The online version of this article (doi:10.1186/s13071-016-1305-z) contains supplementary material, which is available to authorized users.

## Background

In Benin and Burkina Faso, livestock production represents the second contribution after crops to the gross domestic product, without leading to self-sufficiency in animal protein production [1, 2]. In both countries, semi-intensive farming systems and the use of exotic breeds remain exceptional and 95 % of the livestock industry relies on extensive and low-input systems. In Benin, half of the livestock production is concentrated in the north-east where herd rotation among communal pastures, post-harvested crops, savannahs and woodlands optimizes the use of the rare grazing resources [3, 4]. In Burkina Faso, extensive and low-input systems include the transhumant system where part or whole cattle herds move to the south in the dry season and come back to the north in mid-May when the rainy season starts [5]. Traditional farming systems in Burkina Faso also include sedentary systems where cattle, sheep and goats forage together on communal pastures. In such low-input systems, herders cannot afford expensive tick control strategies [6]. As a result, ticks and tick-borne pathogens hamper the development of livestock production in these areas.

The variations in tick biodiversity and abundance on domestic ruminants have been poorly studied in West Africa, with the exception of few surveys performed in Benin or Burkina Faso [7–11]. These studies showed that *Amblyomma variegatum*, a three-host tick that infests cattle and small ruminants, was the native species responsible for the highest economic costs. This species impairs animal growth [12], decreases milk yield [13, 14] and is the vector of *Ehrlichia ruminantium*, a virulent pathogen for sheep and goats that was detected in 10 % of *A. variegatum* adults in several Beninese regions [11]. Three native species of the *Boophilus* subgenus (*R. annulatus*, *R. decoloratus* and *R. geigyi) transmit Babesia bigemina* (the agent of African redwater) *and Anaplasma marginale in this region* [15, 16]. Seven other native species, of little veterinary health concern, were also recorded in these studies: three *Hyalomma* species (*H. impressum*, *H. marginatum rufipes* and *H. truncatum*) and four other *Rhipicephalus* species (*R. muhsamae*, *R. sanguineus, R. senegalensis* and *R. sulcatus*) [7–9]. These surveys also highlighted geographical variations in the predominant species: *A. variegatum* and *H. marginatum rufipes* were the only species found on cattle in Central Burkina Faso [7], while *A. variegatum* and *R. geigyi* represented between 70 and 99 % of the ticks infesting cattle in North Benin [8, 9].

To the best of our knowledge, it is not known whether and how co-infestation patterns influence the abundance of each native tick species. Moreover, the recent invasion of West Africa by the Asian cattle tick *Rhipicephalus* (*Boophilus) microplus* could have modified these tick communities and consequently also the threats to the health of domestic ruminants*. R. microplus* is associated with the highest economic losses where it occurs because of its direct deleterious effects on cattle health and its vector competence for *Babesia bigemina*, *B. bovis* and *A. marginale* [17]. *R. microplus* was introduced in Ivory Coast [18, 19] and Benin [20] in the early 2000s and within a decade it has spread to Togo, Mali, Burkina Faso and along the north-eastern border between Nigeria and Cameroon [21–25]. A nationwide survey performed in Benin found that *R. microplus* was the predominant *Boophilus* species in the southern half of the country in late 2011 [25]. As a consequence, this invasive species was suspected to have outcompeted and replaced its native competitors [19, 25], as it did in South Africa (see [26, 27]). This hypothesis remains nevertheless to be tested. Indeed, as the data from the Beninese survey were expressed in percentages of invasive and native species among the collected ticks, it was not possible to determine whether *R. microplus* invasion has actually decreased the native competitor burden [25]. This was carried out to update the information on tick infestation in domestic ruminants in Benin and Burkina Faso, West Africa, as well as to compare the current abundances of native ticks with those observed before the arrival of *R. microplus*. In addition, the effect of geographical changes on species abundance and tick species assemblages was investigated. To this end, the variations in the composition of tick communities (beta diversity), the contribution of each species and/or each site to the beta diversity and the relative contribution of geographical and climatic variables (mean monthly rainfall and temperature) to the spatio-temporal variations in beta diversity were quantified.

## Methods

### Sampling areas

**Fig. 1:**
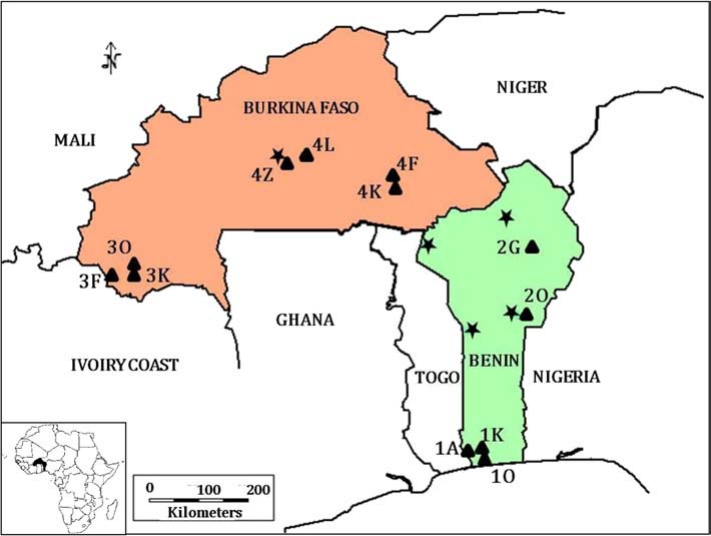
Sampling geography. Sampling sites are represented by triangles and identified by the number of the area followed by the first letter of the locality name. Thus, in area #1 (South Benin), ticks were collected in Athiémé (1A; N 6.5864; E 1.6653), Kpinnou (1 K; N 6.5681; E 1.781) and Ouidah (1O; N 6.3336; E 2.0064). In North Benin (area #2), sampling sites were in Okpara (2O; N 9.305; E 2.7314) and Gogounou (2G; N 10.7383; E 2.9233). In South-West Burkina Faso (area #3), samples were collected in Farnifaso (3 F; N 10.07338; W 4.94975), Kimini (3 K; N 10.07162; W 4.808) and Ouangolodougou (3O; N 10.0858; W 4.77828). In Central Burkina Faso (area #4), sample collection took place in Fada N’gourma (4 F; N 12.05; E 0.35), Kikideni (4 K; N 11.9167; E 0.3833), Loumbila (4 L; N 12.5167; W 1.35) and Zagtouli (4Z; N 12.3167; W 1.6333). Stars indicate the localities where tick abundance on cattle was studied before the arrival of *R. microplus* (one locality from area #4 in 1996 [7]; two localities in the east of area #2 between 2003 and 2004 [9] and two localities in the west of area #2 between 2004 and 2005 [8])

Four areas with different climate were considered (Fig. 1). South Benin has a Guinean climate characterized by a long rainy season from April to July, a short dry season in August, a short rainy season between September and November and a long dry season from December to March. North Benin included two sites where a rainy season (May to October) is followed by a dry season (November to April). Overall, the amount of annual rainfall is 1400 mm in South Benin and 1300 mm in North. In South-West Burkina Faso and Central Burkina Faso the rainy season lasts from June to September and the dry season from October to May. Overall, the amount of maximum rainfall is higher in North Benin (i.e., 1300 mm) than in Burkina Faso (1200 mm) and the most arid area is Central Burkina Faso. These four areas also represent different steps in *R. microplus* invasion of West Africa. The state farm Kpinnou in South Benin (site 1 K, Fig. 1) was the place where the invasive tick was introduced in 2004 [20]. *R. microplus* reached North Benin by 2008 [19, 20] and South-West Burkina Faso in late 2011 [24]. *R. microplus* has never been observed in Central Burkina Faso before the beginning of this study. Two to four herds were monitored in each of these four areas. Hereafter, each sampling site is designated by a number that identifies the geographical area (South Benin: area #1; North Benin: area #2; South-West Burkina Faso: area #3; Central Burkina Faso: area #4) followed by the initial of the site name (e.g., site 1 K corresponds to Kpinnou in South Benin) (Fig. 1).

### Tick sampling

Twelve sentinel steers were randomly chosen within each monitored herd. Sampling started in February 2012 in areas #1 and #2, in April 2012 in area #3 and in May 2012 in area #4. At each of the monthly tick collection events, each sentinel steer was kept with one flank on the ground for 15 min to allow the collection of all the ticks attached on the other half of the body. Ticks were stored in 70 % ethanol. Sampling date, host ID number and attachment site on the host (i.e., head, legs, flank, perineum or tail) were recorded as well as information on the mean monthly rainfall and temperature obtained from ASECNA (Benin) and the “Direction Générale de la Météorologie” (Burkina Faso).

### Tick identification

Although immature ticks were also collected, the analysis focused only on the adult stage to minimize the risks of counting errors [28] and of misidentification within the *Boophilus* sub-genus [16]. Tick identification was performed in two steps: i) identification of *Amblyomma variegatum* ticks and discrimination between the *Hyalomma* genus (hereafter referred to as *Hyalomma spp*), the *Boophilus* subgenus (*Boophilus spp*) and the other *Rhipicephalus* species (*Rhipicephalus spp*), using a stereoscopic microscope at x60 magnification; ii) discrimination of the four *Boophilus* species (i.e., the invasive *R. microplus* species and the three native species *R. annulatus*, *R. decoloratus* and *R. geigyi*) at x100 magnification for more precision, since *Boophilus* species are morphologically very similar. The differentiation criteria were classically based on the number of teeth rows on the hypostome, the form of the male ventral plates as well as the presence (absence) of setae on the internal protuberance of the first segment of palps, of external spur on coxa II and III and of a caudal appendage [16].

### Analysis of the tick community structure and its spatio-temporal variations

The species x locality matrix was computed after Hellinger transformation of the abundance data [29] to estimate the beta diversity (BD), as described in [30]. Such estimate varies between 0 (no geographical variation in species assemblage) and 1 (each surveyed locality hosts a distinct species assemblage). BD was then partitioned into Local Contributions to Beta Diversity (LCBD) or Species Contributions to Beta Diversity (SCBD) [30]. Null LCBD estimates define the null hypothesis of a random distribution of species among localities (i.e., a state where the community occupying any given locality is formed independently from the species assemblages encountered elsewhere) [30]. Significant LCBD deviations from zero were tested by performing 999 random permutations (nperm = 999) of the matrix columns [30]. The largest SCBD estimates are associated with the most heterogeneously distributed taxa, and sites where communities are dominated by species associated with large SCBD estimates tend to display significantly non-null LCBD [30]. The spatio-temporal variations in the community structure and the relative contribution of climatic variables and sampling sites to LCBD variations were investigated as previously described [31]. The correlations between species richness and LCBD estimates were computed to accurately interpret non-null LCBD estimates. A negative correlation is expected when significantly non-null estimates indicate species-poor sampling events [31].

### Tick abundance dynamics

The temporal variations in the abundance of each taxon were analyzed using generalized linear models with a negative binomial structure (i.e., using the glm.nb function from the MASS package in R; http://cran.r-project.org/web/packages/MASS/index.html). In model comparisons, preference was given to models that minimized the Akaike information criterion (AIC), while maximizing the percentage of explained variance. The first step of simplification focused on the seasonal variations in abundance: the months associated with not significantly different (*P* > 0.05) estimates were merged into the same level of the ‘seasonal’ factor s_x_ [32]. The second step tested whether the tick co-infection pattern interacted with s_x_ to determine the abundance dynamics of a given taxon X. *H, A, R, Rm, Ra, Rd* and *Rg* were defined as categorical variables with a value of 1 or 0 to describe the presence or absence of *Hyalomma spp*, *Amblyomma variegatum*, *Rhipicephalus spp*, *R. microplus*, *R. annulatus*, *R. decoloratus* and *R. geigyi*, respectively. In R language, H*A*R*Rm*Ra*Rd*Rg included all additive and interactive effects among these explanatory variables. For simplicity, Π_all-but-x_ defined the term H*A*R*Rm*Ra*Rd*Rg from which the contribution of the taxon X was removed. In R language, the maximal model to explain the abundance variations of taxon X was s_x_ * Π_all-but-x_. Model simplification was achieved by removing the terms without significant effect (*P* > 0.05) on the analyzed abundance.

### Variations in the tick incidence rates

The mean values of the *H, A, R, Rm, Ra, Rd* and *Rg* variables defined above correspond to the incidence rates per steer and per month of *Hyalomma spp*, *Amblyomma variegatum*, *Rhipicephalus spp*, *R. microplus*, *R. annulatus*, *R. decoloratus* and *R. geigyi*, respectively [32]. Their variations among sites (factor SITE), seasons (factor s_x_) and/or hosts with different co-infestation patterns (Π_all-but-x_) were analyzed using generalized linear models with a binomial structure [33]. The maximal model was ~ SITE*s_x_ + Π_all-but-x_. Model simplification was achieved by removing the terms with no significant effect (*P* > 0.05). The possibility of over dispersion (and thus the necessity to perform a new analysis using a quasibinomial model structure) was checked *a posteriori* by computing the ratio of residual deviance onto the residual freedom degrees [31].

### Analysis of tick attachment sites on the host body

Cattle tick species have evolved preferences concerning their attachment sites on the host body: *Rhipicephalus spp* prefers attaching on the head and legs, while *A. variegatum*, *Hyalomma spp* and *Boophilus* ticks favor attachments on trunk and perineum [8, 9, 16]. To investigate the relationships between co-infestation patterns and the distribution of a given tick taxon on the host body, R x C contingency tables were defined in which the C columns describe the distribution of a given tick species across the host body parts (C = 2, when considering the ‘preferred’ vs’not preferred’ body part categories; C = 5, when considering head, legs, flank, perineum and tail as different categories) and the R rows (R = 2) the presence/absence of a co-infesting tick species. Then, the independence between rows and columns was tested using the Fisher’s exact test. Finally, the possibility of a global tendency was tested by combining the obtained *P*-values for a given pair of tick species across sites. Given the low number of *P*-values to combine, the Stouffer’s combination method was preferred [32, 34], using the R process developed by Burns [35].

### Ethics statement

Herders received full information on the study objectives and procedures before signing a written informed consent. Sampling was systematically coupled with veterinary inspection of the herd; in the case of infection, animals received free treatment. All study procedures were reviewed and approved by the CSIRO Social Science Human Research Committee under approval number Ref 038/12.

## Results

### Predominance of the invasive *R. microplus* species

Overall, 144 animals were monitored monthly for one year and 32,148 adult ticks were collected. They all could be identified (genus, subgenus or species), but for 120 specimens (0.37 %). Ticks belonging to the *Amblyomma variegatum* species (*n* = 2,806; 8.76 %), the *Hyalomma* species (*n* = 2,458; 7.67 %) and the *Rhipicephalus* genus, excluding the *Boophilus* subgenus, (*n* = 2,436; 7.60 %) showed a comparable abundance. Ticks belonging to the *Boophilus* subgenus (*n* = 24,328) represented 76 % of the whole collection. Even when taking into account the 842 *Boophilus* ticks that could not be assigned to a species, this subgenus was predominantly represented by the invasive species. Indeed, 22,491 of these ticks were identified as *R. microplus*, 510 as *R. annulatus*, 308 as *R. decoloratus* and 177 as *R. geigyi*.

### Geographical variation in tick assemblages

The overall BD estimate was 0.37. It decreased to ~0.20 and ~0.05, when the data from the four different areas were used separately (Table 1). Area #4 was the only area associated with a significantly non-null LCBD estimate (*P* = 0.013; LCBD ~ 0.68 versus < 0.20 for the other areas). Considering the within-area BD distribution, a significant non-null LCBD estimate was found only at site 4 L (*P* = 0.037, LCBD ~0.64 versus < 0.20 for the other sites). The correlations between species richness and LCBD were significantly negative for the whole dataset (r = -0.57, *P* < 10^-6^), area #2 (r = -0.44, *P* = 0.03) and area #4 (r = -0.35, *P* = 0.01).

**Table 1:** Beta diversity

Parameter		Overall	Sampling areas			
			Area 1	Area 2	Area 3	Area 4
BD		0.37	0.18	0.21	0.053	0.042
SCBD	*H. spp*	0.38	0.0072	0.16	0.21	0.032
	*A. variegatum*	0.0068	0.37	0.32	0.19	0.66
	*R. spp*	0.060	0.40	0.010	0.35	NA
	*R. annulatus*	0.036	0.011	0.059	0.0045	NA
	*R. decoloratus*	0.018	0.024	0.049	NA	0.25
	*R. microplus*	0.491	0.19	0.38	0.23	NA
	*R. geigyi*	0.0031	0.00081	0.016	0.0057	0.061
LCBD		Area 1: 0.17	1A: 0.37	2G: 0.50	3 F: 0.21	4 F: 0.14
		Area 2: 0.08	1 K: 0.43	2 K: 0.50	3 K: 0.61	4 K: 0.03
		Area 3: 0.07	1O: 0.19		3O: 0.19	4 L: 0.65*
		Area 4: 0.68*				4Z: 0.18
Correlation (*P*-value)		-0.57* (<10^-6^)	-0.03 (0.85)	-0.44* (0.03)	-0.09 (0.58)	-0.35* (0.01)

BD, SCBD and LCBD refer to beta diversity, species-contribution to the beta diversity and local-contribution to the beta diversity. ‘NA’ indicates areas where the taxon was absent. The correlation between species richness and LCBD was assessed using the Pearson’s correlation coefficient. Asterisks(*) and bold characters indicate significant(P<0.05) positive correlations

Overall, *R. microplus* (SCBD = 0.49) and *Hyalomma spp* (SCBD = 0.38) showed the highest distribution heterogeneity, while the other taxa were more homogenously distributed (SCBD < 0.04, Table 1). In area #1, *Rhipicephalus spp* and *A. variegatum* (SCBD = 0.40 and 0.37) were the most heterogeneously distributed ticks, followed by *R. microplus* (SCBD = 0.19). In area #2, *R. microplus* and *A. variegatum* (SCBD = 0.38 and 0.32, respectively) showed the highest distribution heterogeneity, followed by *Hyalomma spp* (SCBD = 0.16). In area #3, *Rhipicephalus spp* (SCBD = 0.35), *R. microplus* and *Hyalomma spp* (SCBD = 0.23 and 0.22, respectively) were the most heterogeneously distributed taxa. Area#4 was characterized by high heterogeneity in *A. variegatum* distribution (SCBD = 0.66) and the absence of three taxa (*Rhipicephalus spp*, *R. annulatus* and *R. microplus*).

### Tick assemblage dynamics

**Fig. 2:**
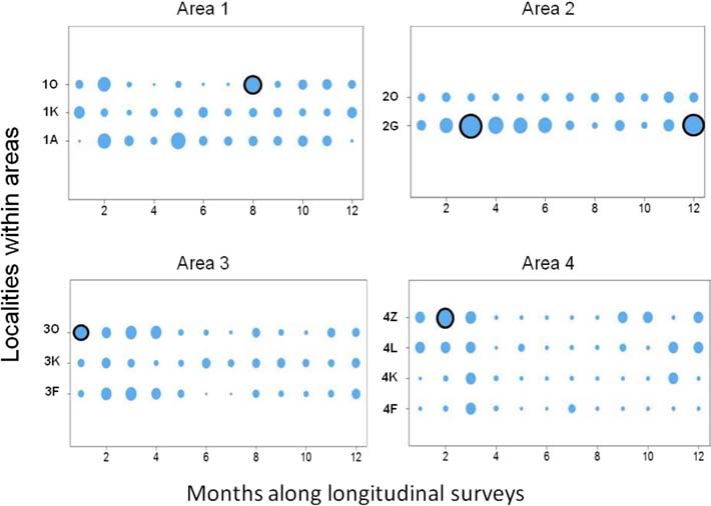
Spatio-temporal LCBD dynamics. The circle size is proportional to the LCBD value. Black rims indicate a significant deviation from the homogeneous distribution (5 % risk; *P* < 0.05). The first sampling month was February 2012 in areas #1 and #2, April 2012 in area #3 and May 2012 in area #4

Within-area LCBD dynamics are detailed in Fig. 2. In area #1, the only significant increase in LCBD was observed at site 1O in September 2012. This sampling event was characterized by a one-off over-representation of *A. variegatum* ticks (48 % of all adults ticks collected in September 2012 compared to 5 % on average in this area). In area #2, significantly higher LCBD values were recorded twice at site 2G. Both were associated with over-representation of one taxon. Specifically, *Hyalomma spp* represented 74 % of all ticks collected in April 2012 (versus 13 % on average in this area) and *R. decoloratus* represented 58 % of all ticks collected in January 2013 (versus 8 % on average). In area #3, a significant LCBD increase was recorded in April 2012 at site 3O. This sampling event was different from the others at site 3O because: (i) *R. microplus,* which was otherwise the most common species in area #3 (75 % of all ticks collected in this area), was absent and (ii) *Hyalomma spp* ticks represented up to 85 % of all sampled ticks (versus 10 % on average). The LCBD values in area #4 were smaller than those recorded in the other areas (Fig. 2). Nevertheless, a significant LCBD increase was observed at site 4Z in June 2012, when *A. variegatum* represented 100 % of all collected ticks (versus 16 % on average).

Variations in the mean rainfall and temperature explained only 3 % of LCBD variations (R^2^_adj-climate_ = 0.03), while the sampling geography explained 54 % of LCBD variations (R^2^_adj-sites_ = 0.54, R^2^_adj-climate_ & _sites joined_ = 0.54).

### Seasonal tick abundance patterns

**Fig. 3:**
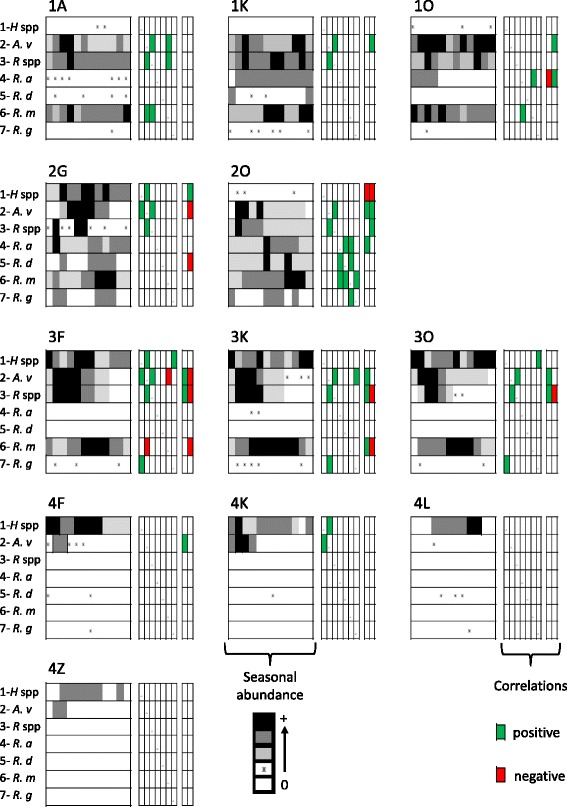
Seasonal variations in abundance. Three panels are shown for each monitored herd/site with the tick taxa listed as follows: *Hyalomma* spp (*H spp*), *A. variegatum (A v)*, *Rhipicephalus* spp (*R spp*), *R. annulatus (R a)*, *R. decoloratus (R d)*, *R. microplus (R m)* and *R. geygyi (R g)*. The first panel illustrates the variation in abundance during the 12 months of the survey (one rectangle for each month; the first sampling month being February 2012 in areas #1 and #2, April 2012 in area #3 and May 2012 in area #4), color-coded as follows: white, absence (empty rectangle) or sporadic (rectangle with an “x”) distribution; light grey, low abundance; dark grey, medium abundance; and black, high abundance of the taxon (see Table 2). The second panel refers to the correlations computed between each possible pair of tick taxa (same ranking order of the taxa). The third panel shows the correlation between the temporal distribution of a taxon and the monthly rainfall (first column) or the monthly temperature (second column). Significant correlations are in green (positive) or in red (negative)

Models failed to converge, and thus to provide seasonal patterns, when all sites of a given area were considered together. Conversely, model convergence, and thus patterns of seasonal variation in abundance, was usually obtained when each site was considered individually. Generally, the abundance of adult ticks of a given species at a given site could be described by a null estimate or at most by three non-null estimate levels (high, medium or low abundance) (Fig. 3 and Table 2). The exception to this rule occurred when a taxon was sporadically present at a site. Such sporadic distribution characterized the three native species of the *Boophilus* subgenus at most sites, but for area #2. Similarly, *Hyalomma spp* was also sporadically present in the most southern sites (Fig. 3).

**Table 2:** Seasonal abundance estimates (number of ticks per host and per month)

Site	Taxon	Seasonal abundance estimates		
		High	Medium	Low
1A	*A. variegatum*	9.3 ± 1.9	2.6 ± 0.6	0.8 ± 0.2
	*Rhipicephalus spp*	18 ± 1.0	6.2 ± 1.0	0.6 ± 0.4
	*R. microplus*	16 ± 4.0	4.8 ± 0.7	0.5 ± 0.2
1 K	*A. variegatum*	0.6 ± 0.2	0.2 ± 0.1	0.04 ± 0.02
	*Rhipicephalus spp*	2.3 ± 0.4	0.7 ± 0.2	0.08 ± 0.08
	*R. annulatus*		0.9 ± 0.2	
	*R. decoloratus*		0.8 ± 0.1	
	*R. microplus*	30 ± 5.5		18 ± 3.7
1O	*A. variegatum*	4.0 ± 0.4	2.5 ± 0.6	0.3 ± 0.3
	*Rhipicephalus spp*		1.4 ± 0.4	0.2 ± 0.07
	*R. annulatus*		3.3 ± 0.2	
	*R. microplus*	15 ± 1.34	8 ± 1.0	1.5 ± 0.7
2G	*Hyalomma spp*	2.9 ± 0.5	0.3 ± 0.2	0.07 ± 0.2
	*A. variegatum*	8.3 ± 1.3	2.7 ± 0.5	0.5 ± 0.1
	*Rhipicephalus spp*		2.6 ± 0.4	
	*R. annulatus*	6.2 ± 3.9	2.8 ± 0.5	0.2 ± 0.1
	*R. decoloratus*		1.9 ± 0.6	
	*R. microplus*	9.2 ± 1.8	2.5 ± 1.0	0.4 ± 0.2
	*R. geigyi*			1.0 ± 0.4
2O	*A. variegatum*	1.5 ± 0.2		0.3 ± 0.1
	*Rhipicephalus spp*	5.5 ± 1.0	1.2 ± 0.3	0.2 ± 0.1
	*R. annulatus*	3.2 ± 1.6	1.3 ± 0.4	0.3 ± 0.1
	*R. decoloratus*	2.3 ± 0.9		0.10 ± 0.05
	*R. microplus*	39 ± 5.5	20 ± 2.5	4.9 ± 1.1
	*R. geigyi*		0.5 ± 0.1	
3 F	*Hyalomma spp*	7.7 ± 1.0	4.6 ± 0.7	2.3 ± 0.8
	*A. variegatum*	10 ± 2.0	2.8 ± 0.7	0.5 ± 0.1
	*Rhipicephalus spp*	7.2 ± 1.2	0.9 ± 0.3	0.06 ± 0.02
	*R. microplus*	34 ± 4.7	5.4 ± 0.8	1.2 ± 0.3
3 K	*Hyalomma spp*	6.8 ± 0.8	3.0 ± 0.5	1.0 ± 0.2
	*A. variegatum*	8.5 ± 1.4		0.5 ± 0.2
	*Rhipicephalus spp*	4.2 ± 0.7		0.2 ± 0.03
	*R. microplus*	98 ± 11	7.3 ± 1.2	1.5 ± 0.5
3O	*Hyalomma spp*	3.5 ± 0.5	1.7 ± 0.2	0.5 ± 0.1
	*A. variegatum*	6.3 ± 1.3	1.0 ± 0.4	0.3 ± 0.1
	*Rhipicephalus spp*	15 ± 1.7	2.3 ± 0.6	1.2 ± 0.1
	*R. microplus*	23 ± 4.2	4.4 ± 0.8	1.1 ± 0.2
4 F	*Hyalomma spp*	3.0 ± 0.5	1.9 ± 0.4	0.7 ± 0.2
	*A. variegatum*		3.0 ± 0.6	
4 K	*Hyalomma spp*	4.3 ± 1.5	1.5 ± 0.3	0.2 ± 0.1
	*A. variegatum*	1.1 ± 0.4		0.1 ± 0.1
4 L	*Hyalomma spp*	1.5 ± 0.3		0.3 ± 0.1
4Z	*Hyalomma spp*		0.4 ± 0.1	
	*A. variegatum*		0.1 ± 0.1	

Analysis of the abundance patterns of *Rhipicephalus spp* and *R. microplus* showed that they were absent in area #4, whereas they were collected in the other three areas all year round (Fig. 3). *Rhipicephalus spp* showed either one long abundance peak or two-three short peaks between March and August (Fig. 3), with the highest abundance level estimates in area #1 (site 1A: 18 ± 0.9 ticks/steer per month) (Table 2). *R. microplus* abundance showed several uncoordinated peaks in the herds from area #1, but peaked once per year in the other areas where it was observed (from September to December in area #2, and from July to January in area #3, Fig. 3). Its monthly abundance reached 98 ± 11 ticks/steer at site 3 K, but remained below 50 ticks/steer elsewhere (Table 2).

*Hyalomma spp* abundance peaked twice during the year (from February to June and from August to December), with adults collected all year around except in two of the four sites of area #4 (4 L and 4Z). During the high abundance season, abundance estimates were highest in area #3 (site 3 F: 7.7 ± 1.0 ticks/steer per month) and lowest in area #4 (site 4 K: 4.3 ± 1.5 ticks/steer per month) (Table 2).

*A. variegatum* adults were generally collected everywhere and all year round. Its abundance tended to peak once in the most arid sites and twice in area #1 (Fig. 3), with the highest estimates in area #3 (site 3 F: 10.1 ± 2.0 ticks/steer per month) (Table 2).

Congruence in the seasonal variation patterns was observed between *A. variegatum* and *Rhipicephalus spp* and their burdens were significantly and positively correlated at seven of the eight sites were they were both present (Fig. 3; combined *P*-value across herds: *P* = 8. 10^-36^). For these two species, abundance peaks were observed at the beginning of the rainy season (i.e., in April-May in area #1, May-June in area #2 and March-April in area #3) and their abundance dynamics were positively correlated with the rainfall variations (Fig. 3; *A. variegatum*: five significantly positive correlations; combined *P*-values across herds: *P* = 5. 10^-11^; *Rhipicephalus spp*: three significantly positive correlations; combined *P*-value across herds: *P* = 3. 10^-20^). Similarly, *A. variegatum* and *Hyalomma spp* abundance dynamics were positively correlated (Fig. 3; three significantly positive correlations; combined *P*-value: *P* = 9. 10^-13^). Differently from these native ticks, *R. microplus* abundance peaked a few months after the beginning of the rainy season. This resulted in a significantly negative correlation between *A. variegatum* and *R. microplus* abundance dynamics in area #3 (Fig. 3; site 3 F). Conversely, significantly positive correlations were detected between the abundance of the native *Boophilus* species and that of *R. microplus* in area #2 (Fig. 3; site 2O), although the small number of native ticks weakened the statistical power of the analysis.

### Effect of co-infestation patterns on adult tick abundances and incidence rates

Besides seasonality, the host co-infestation pattern also significantly structured the within-site abundance variations in 19 of the 33 (57 %) taxon-by-site combinations defined by the four predominant taxa (*Hyalomma spp*, *A. variegatum*, *Rhipicephalus spp* and *R. microplus*).

**Fig. 4:**
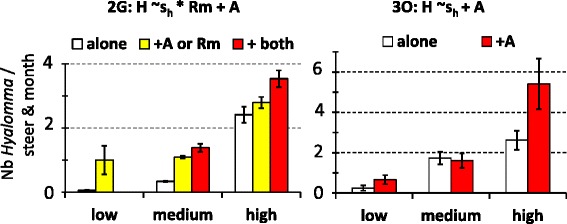
Significant impacts of the host co-infestation pattern in *Hyalomma spp* abundance. The minimal models (i.e., involving factors with significant effects; *P* < 0.05) are indicated: s_h_ refers to the seasonal abundance variations of *Hyalomma* spp while *A* and *Rm* describe the presence or absence of co-infestation by *A. variegatum* and *R. microplus*, respectively. The histograms refer to the observed distributions. The indications ‘alone’, ‘+X’ or ‘+ both’ refer to the absence of competitors on the individual-host, or the presence of one or both co-infesting taxa, respectively

Significant effects of competitors were less frequently observed on *Hyalomma spp* abundance than on other tested taxa (two significant interactions out of seven tested sites; 28 % *vs* ≥ 50 % for the other taxa). Specifically, *Hyalomma spp* abundance at site 2G during the medium and high abundance seasons was significantly (*P* < 0.05) higher in the case of co-infestation by *A. variegatum* and *R. microplus* than in the absence of co-infestation (Fig. 4). A similar, but more pronounced effect of co-infestation by *A. variegatum* on *Hyalomma spp* abundance was observed at site 3O during the low and high abundance season (Fig. 4).

**Fig. 5:**
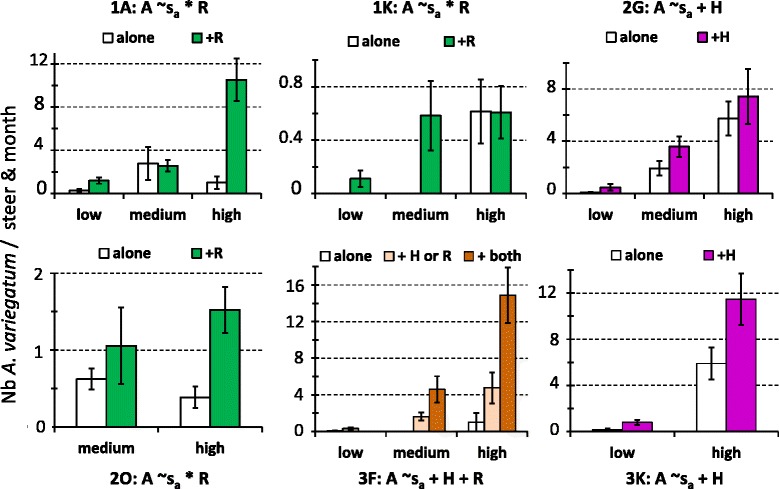
Significant impacts of the host co-infestation pattern in *A. variegatum* abundance. The minimal models (i.e., involving factors with significant effects) are indicated: s_a_ refers to the seasonal abundance variations of *A. variegatum* while *H* and *R* describe the presence or absence of co-infestation by *Hyalomma* spp and *Rhipicephalus spp*, respectively. The histograms refer to the observed distributions (see Fig. 4 legend)

Similarly, *A. variegatum* abundance significantly increased on hosts that were co-infested by *Hyalomma spp* at sites 2G, 3 K and 3 F (Fig. 5). At site 3 F, this effect was further increased when the host was simultaneously co-infected with *Rhipicephalus spp* and *Hyalomma spp* (Fig. 5). At three other sites (1A, 1 K and 2O), host co-infestation by *Rhipicephalus spp* also had a season-dependent, positive effect on *A. variegatum* abundance (Fig. 5), with some exceptions. Specifically, *Rhipicephalus spp* co-infestation had no significant effect at sites 1A and 2O in the medium abundance seasons (Fig. 5).

**Fig. 6:**
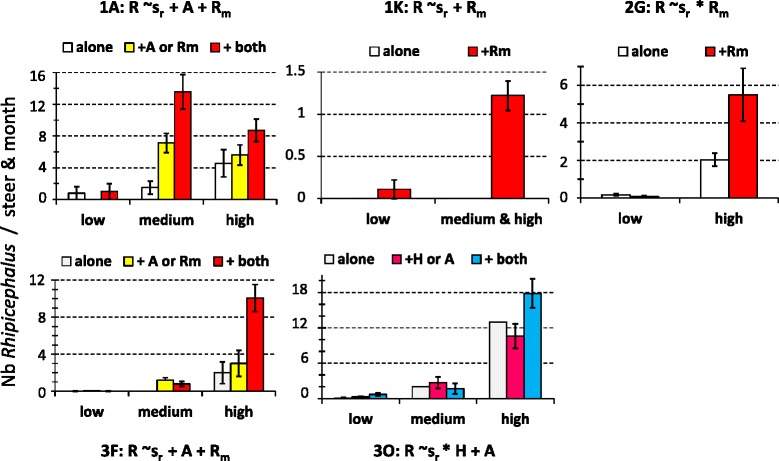
Significant impacts of the host co-infestation pattern in *Rhipicephalus spp* abundance. The minimal models (i.e., involving factors with significant effects) are indicated: s_R_ refers to the seasonal abundance variations of *A. variegatum* while *A*, *H* and *Rm* describe the presence or absence of co-infestation by *A. variegatum*, *Hyalomma* spp and *R. microplus*, respectively. The histograms refer to the observed distributions (see Fig. 4 legend)

Analysis of the co-infestation effect on *Rhipicephalus spp* abundance indicated that the presence of *A. variegatum* and/or *R. microplus* increased *Rhipicephalus spp* abundance in five of the eight herds where it was found (sites 1A, 1 K, 2G, 3 F and 3O) (Fig. 6). Conversely, at 2G, the season-dependent effect of co-infestation by *R. microplus* resulted in a decrease of *Rhipicephalus spp* abundance in the low season (from 0.2 to 0.1 tick/steer per month) and in an increase in the high season (from 2 to 5.5 ticks/steer per month; Fig. 6).

**Fig. 7:**
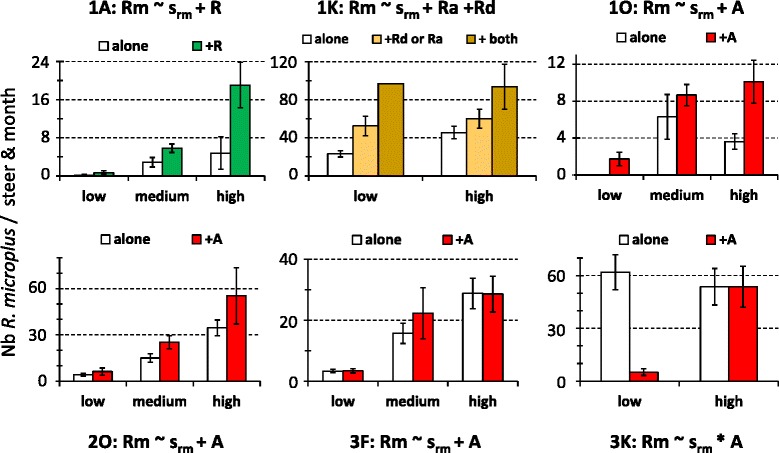
Significant impacts of the host co-infestation pattern in *R. microplus* abundance. The minimal models (i.e., involving factors with significant effects) are indicated: s_Rm_ refers to the seasonal abundance variations of *A. variegatum* while *A*, *R, Ra* and *Rd* describe the presence or absence of co-infestation by *A. variegatum*, *Rhipicephalus* spp, *R. annulatus* and *R. decoloratus*, respectively. The histograms refer to the observed distributions (see Fig. 4 legend)

Finally, *R. microplus* abundance significantly changed with co-infestation by native tick taxa in six out of eight sites (Fig. 7). Co-infestation by *A. variegatum* significantly increased *R. microplus* abundance at sites 1O and 2O in all seasons and at site 3 F in the medium abundance season (Fig. 7). Conversely, at site 3 K, *A. variegatum* co-infestation decreased *R. microplus* monthly abundance from 62 to 5.1 ticks/steer in the low abundance season (Fig. 7). Three other native taxa significantly (*P* < 0.05) increased *R. microplus* abundance. At site 1A, co-infestation by *Rhipicephalus* spp increased *R. microplus* abundance in all abundance seasons (Fig. 7). At site 1 K, co-infestation by *R. annulatus* and/or *R. decoloratus* increased *R. microplus* abundance during the low and high abundance seasons (Fig. 7).

**Fig. 8:**
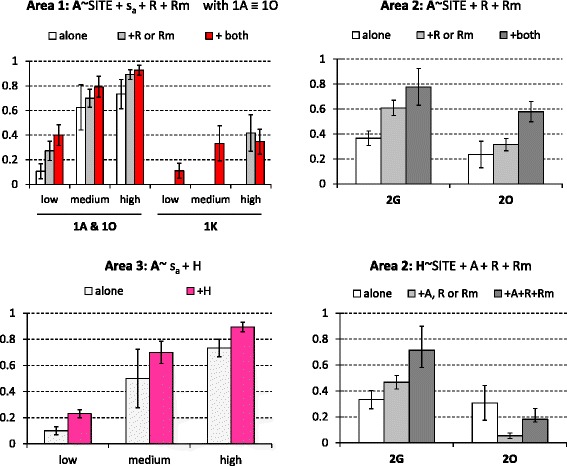
Within-area variations in incidence rates for *A. variegatum* and *Hyalomma spp*. The minimal models (i.e., involving factors with significant effects) are indicated. The histograms refer to the observed distribution in incidence rates among sites, seasons and/or co-infestation patterns

**Fig. 9:**
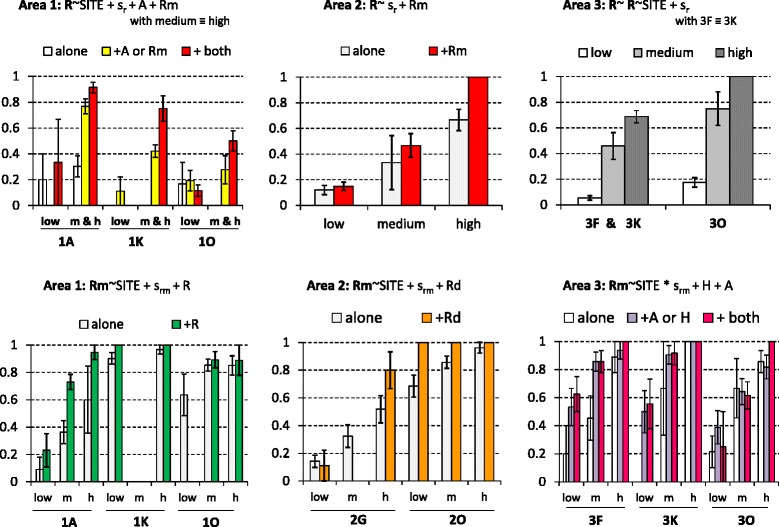
Structure of the within-area variations in incidence rates for *Rhipicephalus spp* and *R. microplus*. The minimal models (i.e., involving factors with significant effects) are indicated. The histograms refer to the observed distribution in incidence rates among sites, seasons and/or co-infestation patterns

The minimal models retained to explain the within-areas of the tick incidence rates involve the additive and/or interactive significant effects of sites, seasonal pattern of abundance and co-infestation patterns. The minimal models retained for *A. variegatum* in area #4 and *Hyalomma* spp in areas # 3 and 4 were not considered since they explained less than 10 % of the variation in the tick incidence rates. The ten others are presented with the observed variations in incidence rates in Figs. 8 and 9. In eight cases, the incidence rates of the studied taxon significantly increased with its seasonal abundance levels (Figs. 8 and 9). Besides seasonality, the incidence rate of *Hyalomma* spp significantly increased upon co-infestation by three other taxa in site 2G. Similarly, the incidence rate of *A. variegatum* significantly increased upon co-infestation by *Rhipicephalus spp* and *R. microplus* in area #1 and #2 and upon co-infestation by *Hyalomma spp* in area #3 (Fig. 8) Co-infestation by *A. variegatum* and/or *R. microplus* increased *Rhipicephalus spp* incidence rate also in area #1 (sites 1A, 1 K and 1O) in the high abundance season, and in area #2 in all three abundance seasons (Fig. 9).

*R. microplus* incidence rates also significantly (*P* < 0.05) increased with co-infestation by *Rhipicephalus spp* in area #1, by *R. decoloratus* in area #2 and by both *A. variegatum* and *Hyalomma spp* in area #3 (Fig. 9). Such effect was particularly visible at site 2O, where *R. microplus* incidence rate reached 100 % among hosts already infested by *R. decoloratus,* irrespective of the season (Fig. 9). *R. microplus* incidence rates of 100 % (high abundance season) were also observed in the three sites of area #3 only in hosts co-infested by both *A. variegatum* and *Hyalomma spp* (Fig. 9).

### Impact of co-infestation patterns on tick distribution at attachment-sites

Analysis of the effect of co-infestation by the four predominant taxa (*Hyalomma spp, A. variegatum, Rhipicephalus spp* or *R. microplus*) indicated that the presence of competitors often affected the distribution of other ticks on the five host body parts considered (head, legs, flanks, perineum and tail) (see for details the distribution in *P*1-values in Additional file 1). Specifically, co-infestation by *Hyalomma spp* significantly affected the distribution of *A. variegatum, R. microplus* and *R. geigyi microplus* (in all cases, combined *P*-value across herds < 0.05). Co-infestation by *A. variegatum* significantly affected the distribution of *Rhipicephalus spp, R. annulatus, R. decoloratus* and *R. microplus* (in all cases, combined *P*-value across herds < 0.05). Co-infestation by *Rhipicephalus spp* significantly affected the distribution of all other taxa (in all cases, combined *P*-value across herds < 0.02) and co-infestation by *R. microplus* significantly affected the distribution of all other taxa (combined *P*-value across herds < 0.05), but for *Rhipicephalus spp* (combined *P*-value across herds *P* = 1).

However, such effects rarely influenced the probability of a taxon to reach its favorite attachment sites (see the distribution in *P*2-values in Additional file 1). Only *R. microplus* showed a significant decrease in the probability of reaching its favorite attachment sites across the monitored herds upon host co-infestation by *Hyalomma spp* or *A. variegatum* (in both cases, combined *P*-value across herds < 0.05). Similarly, co-infestation by *Rhipicephalus spp* decreased the probability of *R. microplus* and *R. geigyi* to reach their favorite attachment sites (in both cases, combined *P*-value across herds *P* < 0.05), whereas co-infestation by *R. microplus* decreased the probability of *R. annulatus* to reach its favorite attachment sites (combined *P*-value across herds *P* < 0.05).

## Discussion

This study investigated the determinants in the community structure of ticks infesting cattle in Benin and Burkina Faso. The tick community compositions varied significantly both within and among areas and the lowest diversity was observed in Central Burkina Faso (area #4) (Table 1; Fig. 2). Climatic differences are likely to explain the between-areas variations but not the within-areas variations, given that the variations in the mean rainfall and temperature explained only 3 % of the spatio-temporal LCBD variation. The alternation of dry and rainy seasons represents nevertheless a factor structuring the significant increases in LCBD punctually observed since they occurred in the early rainy season in Benin and South-West Burkina Faso (April- June), in the late rainy season in South Benin (September) and during the early dry season (December) in North Benin (Fig. 2). Furthermore, tick abundance tended to peak during the rainy seasons, although some delay was observed for the *Boophilus* species relatively to the other taxa. Such delay is related to the life cycle specificity of this one-host tick species. Eggs and unfed larvae are the only stages of the *Boophilus* species living away from the host. Conversely, the other taxa are two- and three-host ticks and not only the eggs and freshly hatched larvae but also other stages can leave the host after complete blood-feeding, thus facing the risk of desiccation in the local habitat [16]. Whatever their life cycle and status (native or invasive species), two infestation dynamic features were common to all tick taxa. First, their infestation dynamics were determined not only by abiotic parameters (seasonal patterns, Table 1), but also by inter-species interactions among cattle ticks (Figs. 4, 5, 6 and 7). Second, positive relationships were observed between their local abundance and incidence rate (Figs. 8 and 9); in other words, the tick probability to infect a new host increases with its local mean abundance. This reminds the positive relationships between local mean abundance and prevalence previously reported for fleas [36], nematodes [37] or monogeneans [38].

In Central Burkina Faso (area #4), *A. variegatum* and *Hyalomma spp* were the only adult ticks collected. Differently from what observed in other taxon-by-climatic area combinations, their seasonal abundance variation was not affected by the host co-infestation pattern in this area. In a previous survey performed in 1996 in three cattle herds from Central Burkina Faso, the monthly abundances of *H. marginatum rufipes* (the only *Hyalomma* species observed) and *A. variegatum* were estimated to be 7.21 and 7.50 adult ticks/steer (Table 3) [8]. Such estimates are higher than those of the present survey (1.22 and 0.73 adult ticks/steer per month) (Table 3), indicating a decrease in the abundance of native ticks during the last decades in an area not colonized by *R. microplus*. Such a decrease might be the result of global warming in this Sahelian region located along the limit of the geographical distribution of these species [16]. Moreover, the recycling for tick control of the chemicals designed for agricultural pest control [6] might also have partly contributed to such a decrease.

**Table 3:** Temporal changes in the abundance of native tick taxa

Year	site	*Hyalomma spp*	*A. variegatum*	*Rhipicephalus spp*	*R. annulatus*	*R. decoloratus*	*R. geigyi*	*Boophilus spp*	All taxa
North Benin (area #2)									
2003–04	All sites	0.52 ± 0.08	1.19 ± 0.20	4.04 ± 0.12	0.31 ± 0.07		1.15 ± 0.17	1.46 ± 0.20	3.50 ± 0.47
2004–05	All sites	0.40 ± 0.14	4.13 ± 1.33	0.12 ± 0.03	0.19 ± 0.08		1.07 ± 0.17	1.26 ± 0.17	5.92 ± 1.50
	Bassila	0.14 ± 0.04	4.59 ± 0.22	0.12 ± 0.04	0.12 ± 0.04		1.12 ± 0.18	1.28 ± 0.22	6.13 ± 1.49
	Materi	0.67 ± 0.27	3.68 ± 0.16	0.34 ± 0.12	0.21 ± 0.06		1.02 ± 0.02	1.23 ± 0.17	5.71 ± 1.58
2012–13	All sites	0.79 ± 0.21	2.02 ± 0.81	0.83 ± 0.22	0.72 ± 0.28	1.12 ± 0.33	0.29 ± 0.11	2.13 ± 0.65	5.77 ± 0.92
	2G	1.47 ± 0.40	3.33 ± 0.78	0.76 ± 0.35	0.93 ± 0.51	1.34 ± 0.55	0.35 ± 0.18	2.63 ± 0.78	8.18 ± 1.83
	2O	0.24 ± 0.16	0.74 ± 0.50	0.91 ± 0.44	0.50 ± 0.28	0.85 ± 0.27	0.22 ± 0.09	1.58 ± 0.55	3.47 ± 0.47
Central Burkina Faso (area #4)									
1996	All sites	7.21 ± 1.54	7.50 ± 4.14						7.35 ± 2.59
2012–13	All sites	1.22 ± 0.26	0.73 ± 0.17						0.97 ± 0.21
	4 F	1.94 ± 0.50	0.53 ± 0.33						1.24 ± 0.34
	4 K	1.26 ± 0.35	0.17 ± 0.14						0.72 ± 0.23
	4 L	0.66 ± 0.28	0.007 ± 0.007						0.33 ± 0.14
	4Z	0.23 ± 0.12	0.056 ± 0.037						0.14 ± 0.06

The mean monthly abundance of adult ticks/steer were computed from the data collected in 1996 [7], 2003–04 [9], 2004–05 [8] and 2012–13 (present study); the standard error of the mean refers to the variations observed during the 12 months of each survey. *Boophilus spp* includes the three native species of this subgenus (*R. annulatus, R. decoloratus* and *R. geigyi*)

Although absent from Central Burkina Faso (area #4), *R. microplu*s represented 70 % of all the adult ticks collected in this survey. This confirms the invasion success of *R. microplu*s in West Africa [17–25] and further supports the hypothesis that the Sahelian climate is not suitable to *R. microplu*s [33], although this species was detected along the Cameroon-Nigeria border [22, 23]. Moreover, the high *R. microplu*s abundance in North Benin (area #2) suggests that the presumed ecological niche of this species in West Africa [39] should be re-evaluated. The analysis of the tick attachment sites on the host indicated that *R. microplu*s success to reach its favorite attachment sites significantly decreased upon co-infestation by *Hyalomma spp* and *Rhipicephalus spp.* Conversely, co-infestation by *R. microplu*s did not affect the success of native tick species to attach on their favorite sites (but for *R. annulatus*) (Additional file 1). As the favorite attachment sites are likely to be the result of evolution, these results suggest that the native tick species impose higher competitive constraints on the invasive species than the invasive species on the native ones. Experimental manipulation of host co-infestation and tick fertility monitoring would be required to settle this point. However, the competition exerted by *R. microplu*s on native tick species can be assessed by comparing the present abundance estimates with those obtained before *R. microplu*s arrival [7–9]. The surveys performed in North Benin in the early 2000s led to different estimates in the adult abundance of the native tick taxa than the present study (Table 3). Interestingly, *R. decoloratus* was not detected in these earlier studies, while our survey found that it was the predominant native species of the *Boophilus* sub-genus. Moreover, the abundance of *R. geigyi* adults has decreased since the early 2000s, whereas the abundance of *R. annulatus* adults has increased (Table 3). Overall, the abundance of adult ticks from the native *Boophilus* species (*R. decoloratus*, *R. geygyi* and *R. annulatus*) has increased from 2003 to 2013 (Table 3). This does not support the hypothesis that *R. microplus* is replacing the native sister-species. The same conclusion is reached when considering all native tick taxa (Table 3). The composition of the tick communities has changed over the years; however these changes do not translate in an overall decrease in the abundance of native taxa. Therefore, rather than displacing the native tick taxa and the associated veterinary health concerns, the successful demographic increase of *R. microplu*s in West Africa has added new tick-associated risks for cattle health.

Aggregated distributions of tick taxa among herds were recurrently observed in Benin and South-West Burkina Faso (areas #1 to 3) because the local abundances and/or incidence rates of each taxon increased with the co-infestation by other taxa. This is a characteristic shared by several mammalian ectoparasite communities [39–43]. As genetic bases for this trait exist in cattle, this may open the road to the selection of breeds with lower susceptibility to tick infestation [17]. This feature may also facilitate the communication to local stakeholders regarding tick control. Indeed, the economic advantage of focusing tick control programs on animals that are infested above a threshold burden might counter-balance the average loss in animal growth and milk yield in places where cattle production rely mainly on low input systems and tick burden remains moderate, such as in West Africa. It remains to determine whether the aggregated distribution of tick taxa also affects the distribution of the tick-borne pathogens circulating among West African cattle. Variable patterns have been previously reported. Competitive exclusion among tick-borne pathogens was observed in Algerian cattle [44]. Conversely, aggregative distribution seems to be the rule in South Africa for tick-borne pathogens monitored in mammalian blood samples or in the tick salivary glands [45, 46].

## Conclusion

This study is the first attempt to quantify the impact of co-infestation patterns among native and invasive tick species. It shows that these patterns are key factors in the determination of the infestation dynamics of each tick taxon in West African cattle. It also shows that the *R. microplus*-related risks for cattle health in West Africa are not replacing but are adding to those caused by native tick taxa.

## Additional file

Additional file 1:
**Effect of the co-infestation patterns on the distribution of tick attachment sites.** (XLS 98 kb)
